# The Diagnosis and Significance of Enlarged Mediastinal Glands

**Published:** 1908-05-16

**Authors:** C. Willett Cunnington


### The General Practitioner's Column.

[Contributions to this Column are invited, and if accepted will be paid for.]

### THE DIAGNOSIS AND SIGNIFICANCE OF ENLARGED MEDIASTINAL GLANDS.

By C. WILLETT CUNNINGTON, M.B., B.C.

This is a subject but scantily dealt with in most text-books, although consideration suggests that it is of great importance. The explanation of this omission is probably due to the difficulty hitherto experienced in ascertaining, with any degree of certainty, the condition of the lymphatic glands in the chest. Modern methods lead one to suppose that their enlargement is of very common occurrence.

What are the physical signs of enlarged mediastinal glands? They are chiefly those of pressure: in moderate enlargement, pressure on bronchi; in great enlargement, pressure on veins. Hence, we look for deficient expansion of one side of the thorax, accompanied by weak breath sounds; in more marked cases we may detect enlarged veins coursing over the upper part of the chest; while occasionally Eustace Smith's sign, a venous hum over the angle of Ludovic when the patient's neck is over-extended, may be heard, indicating pressure on the great veins in the root of the neck.

Yet it is seldom that such physical signs are conspicuous, nor do they indicate more than the existence of some pressure; they do not distinguish, for instance, between aneurysmal, glandular, or neoplastic pressure. Moreover, the diagnosis should be made before this pressure has become sufficiently marked to give rise to the physical signs described. For this purpose the patient should be examined by the *x*-rays. A photograph in such cases is often useful, but it is simpler, and generally sufficient, to work with the fluorescent screen. Quite a "soft" tube should be used, lest the rays pass through the glands undiminished in intensity, and cast no shadow.

We look for an increase in size and density of the shadow at the site of the lung-root, with the rays passing antero-posteriorly, noting also any diminution of diaphragmatic excursion. Then the patient is examined in the left oblique position, so that the rays pass in a direction roughly corresponding to a line penetrating from the right scapula behind to the left nipple in front. To examine him in the "right oblique" he is turned so that the rays enter the back a little to the left, and emerge in front on the right of the middle line.

The first manœuvre gives a picture of the anterior and superior mediastina, while the second gives that of the posterior mediastinum. The latter is the more important. Here we see a light space, the "retro-cardiac triangle," bounded by the heart in front, the diaphragm below, and the spine behind. On deep inspiration a streak of light extends upwards behind the heart, and this is the posterior mediastinal space. If the glands are enlarged this space is partially or completely obscured. With a little practice each mediastinal space can readily be examined and any glandular enlargement discovered.

The significance of such enlargement lies in the fact that tuberculosis of the lungs commonly begins by extension from the lymphatics at the lung-root. We may pick out patients who have already in their chests the germs that may ultimately lead to "consumption," and we are able thereby to take suitable precautions. We should not wait for "physical signs." It is common to find slight glandular enlargement in children after the specific fevers; this is not necessarily tuberculous, but it may take months before the glands completely subside.

It is unnecessary to refer to diseases of the type of lymphadenoma, where the enlargement is often very marked. I would like, however, to call attention to the importance of this *x*-ray examination in all cases of *carcinoma of the breast*, before the patients are submitted to operation. Clearly if such a patient has already glandular enlargement in the mediastina, an extensive operation is futile. No surgeon omits to examine the axillary glands before operating, but I venture to urge that in future his investigations should not rest there. The mediastinal glands must also be examined, and this can only be done satisfactorily by the *x*-rays.

